# Bispecific Antibodies and Antibody–Drug Conjugates in Advanced Gastric Adenocarcinoma

**DOI:** 10.3390/cancers18091448

**Published:** 2026-04-30

**Authors:** Jane E. Rogers, Jaffer A. Ajani

**Affiliations:** 1 Department of Pharmacy Clinical Services, University of Texas M. D. Anderson Cancer Center, 1515 Holcombe Blvd, Houston, TX 77030, USA; 2 Department of Gastrointesintal Medical Oncology, University of Texas M. D. Anderson Cancer Center, 1515 Holcombe Blvd, Houston, TX 77030, USA

**Keywords:** gastric adenocarcinoma, gastroesophageal junction adenocarcinoma, zanidatamab, human epidermal growth factor receptor-2, claudin 18.2, trophoblast cell-surface antigen 2, microsatellite instability-high

## Abstract

Advanced gastric (GAC) or gastroesophageal junction (GEJAC) adenocarcinoma continues to carry a poor prognosis. Understanding GAC/GEJAC at the molecular level has provided a new understanding and the basis for individualized approaches to treatment. The current biomarker-driven therapy focuses on four areas: microsatellite instability (MSI), human epidermal growth factor receptor-2 (HER2), programmed death ligand-1 (PD-L1) combined positive score, and claudin 18.2 (CLDN18.2). However, because of improving technology, the focus has shifted to cancer cell-surface proteins and peptides. Each of these GAC/GEJAC subgroups provides a different treatment pathway. The agents utilized to treat advanced GAC/GEJAC include immune checkpoint inhibitors (ICIs), chemotherapy, monoclonal antibodies (mAbs), and antibody–drug conjugate (ADC) therapy, as well as bispecific antibodies (BsAbs), but they are certainly not limited to the above. Drug development has shifted in recent years to establish different mechanisms that are attempting more sophisticated and targeted approaches, such as BsAbs and ADCs. Meanwhile, the development of cytotoxics has tapered off. Along with these developments in drug therapy, more therapies directed at CLDN18.2, HER2, MSI, EGFR, HER3 and trophoblast cell-surface antigen 2 (TROP2) are underway. Here we review future areas in advanced GAC, including zanidatamab’s potential role in HER2-positive advanced GAC and deciphering the abundance of anti-CLDN18.2, extending beyond investigative therapies.

## Simple Summary

Advanced gastric or gastroesophageal junction cancer should be managed using individualized biomarker-based treatment approaches in light of recent findings. New therapies and targets are being investigated, including a focus on certain proteins, like claudin 18.2 and trophoblast cell-surface antigen 2. In addition to new targets, there are new approaches being developed that can address well-known targets in gastric or gastroesophageal junction cancer, like human epidermal growth factor receptor-2 and microsatellite instability-high. The field is growing rapidly. Here we discuss upcoming therapies for gastric or gastroesophageal junction cancer.

## 1. Introduction

Gastric adenocarcinoma (GAC) is the fifth most frequently diagnosed malignancy and the fifth leading cause of cancer death worldwide [1]. GAC is most prevalent in East Asian and South American countries. By some accounts, GAC is on the rise globally and is affecting younger individuals [2,3]. Although the incidence is declining in the United States and Western countries, GAC remains a relevant concern [1,4]. GAC diagnosed in these countries will often be in the advanced setting (non-surgical candidates), making prognosis poor. This advanced diagnosis is due to a lack of routine screening in these geographical areas. The National Cancer Institute (NCI) Surveillance, Epidemiology, and End Results (SEER) program reports a 5-year relative survival of 7.5% for those with distant spread [4]. GAC has historically been classified based on anatomic location (proximal or distal) and histology (intestinal type or diffuse type), with few differences in treatment based on these traditional classifications [1]. In 2014, The Cancer Genome Atlas (TCGA) defined GAC at the molecular level, providing new insights and potential individualized treatment approaches [5]. The TCGA GAC (STAD) classifications included Epstein–Barr virus-positive (8.8%), microsatellite instability-high/deficient mismatch repair (MSI-H/dMMR) (21.7%), genomically stable (19.7%), and chromosomal instability (49.8%). These categorical distinctions help with trial design and enable more individualized treatment approaches.

Advanced gastroesophageal junction adenocarcinoma (GEJAC) is often treated and investigated similarly to advanced GAC [6]. GEJAC represents tumors located between the stomach and esophagus (ranging from 5 cm above the GEJ to 5 cm below the GEJ). Traditional classification is based anatomically on the location relative to the GEJ: Siewert Type I (1 to 5 cm above the GEJ), Siewert Type II (1 cm above to 2 cm below the GEJ), or Siewert Type III (2 to 5 cm below the GEJ). However, in the advanced setting, these anatomic distinctions matter little when deciding treatment as adenocarcinomas are generally managed similarly. The TCGA results for GEJAC identified three proteomic subtypes and potential druggable targets [7].

Biomarker-driven therapy is becoming a staple in oncology practice. Although not as robust as in other solid malignancies, GAC has witnessed substantial development in the biomarker arena in recent years. Patients with advanced GAC/GEJAC are recommended to undergo molecular testing at diagnosis. Currently, this comprises tissue-based testing. Molecular testing includes MSI-H/dMMR, human epidermal growth factor receptor-2 (HER2), programmed death ligand-1 (PD-L1) combined positive score (CPS), and claudin 18.2 (CLDN18.2) [1]. Results help to refine treatment. MSI-H/dMMR patients (<5% of cases) are treated with immune checkpoint inhibitor (ICI) therapy unless it is contraindicated [1,8]. HER2-positive GAC/GEJAC (~15% of cases) is defined as HER2 protein 3+ staining by immunohistochemistry (IHC) or HER2 protein 2+ staining by IHC with an ERBB2/CEP17 ratio ≥2 using fluorescence in situ hybridization (FISH), or an average of ERBB2 copy number ≥6 signals/cell [1]. These HER2-positive tumor patients have options for HER2-directed therapy via front-line trastuzumab plus chemotherapy (platinum + fluoropyrimidine) +/− pembrolizumab followed by trastuzumab deruxtecan in the second-line setting. PD-L1 CPS, which is defined as the number of PD-L1-stained cells (tumor cells, lymphocytes, and macrophages) divided by the total number of viable tumor cells evaluated multiplied by 100, is an additional GAC biomarker [1]. Those with PD-L1 CPS ≥ 5 (~60% of cases) are treated with anti-PD-1 therapy, such as nivolumab/pembrolizumab/tislelizumab, with upfront chemotherapy (platinum plus fluoropyrimidine) [1,9]. CLDN18.2 is the newest GAC biomarker/target [1]. Currently, zolbetuximab, an anti-CLDN18.2 monoclonal antibody (mAb), is approved in combination with upfront fluoropyrimidine plus platinum for those with >75% of viable tumor cells (2+ or 3+ intensity) [10]. This percentage of positivity is reported to occur in ~30% of GAC/GEJAC patients [1,11,12]. However, studies are exploring newer CLDN18.2-targeted agents at lower labeling indices. The overlap of these molecular markers is still being defined.

## 2. Current and Future Outlooks Based on GAC Subtypes

### 2.1. MSI-H-dMMR GAC: Current Treatments and Future Outlooks

#### First-Line Therapy

MSI-H/dMMR is reported to account for ~5–12% of GAC cases, but only ~4% are MSI-H/dMMR in the advanced GAC setting [9,13,14,15]. In early investigations of colorectal cancer (CRC) and solid tumors, patients with MSI-H/dMMR cancers have significantly different outcomes with immune checkpoint therapy compared to MSI-stable/proficient MMR patients [16,17]. Although no dedicated phase 3 trials in MSI-H/dMMR advanced GAC/GEJAC have been performed, these patients are recommended upfront immune checkpoint therapy with either immune checkpoint monotherapy anti-programmed death-1 (anti-PD-1) mAbs or dual immune checkpoint therapy with anti-PD-1 plus anti-cytotoxic T-lymphocyte-associated protein-4 (anti-CTLA4) mAbs [1]. These recommendations come from extrapolating results from phase 1 and phase 2 trials and sub-analyses of large phase 3 front-line trials. NCT06346197 aims to answer which therapy is preferred [18]. NCT06346197 is a phase 3 trial exploring the combination of botensilimab, an anti-cytotoxic T lymphocyte-associated protein 4 (CTLA-4) antibody, plus balstilimab, an anti-PD-1 antibody, compared to nivolumab + SOC chemotherapy. Estimated completion is in 2028. Remarkable outcomes in MSI-H/dMMR GAC have been noted in these mAb results, evidenced by reports of 59% pathological complete responses (pCRs) in localized disease and prolonged survival in metastatic GAC [1]. pCRs in the general GAC population with traditional chemotherapy (5-fluorouracil + oxaliplatin + docetaxel) are reported at ~16%. However, many patients experienced relapse. Some respond to cytotoxic combinations but will need more sophisticated approaches, such as bispecific antibodies (BsAbs) or ADCs, as advances in antibody engineering continue to improve upon these single-pathway-inhibiting (monospecific) mAbs [19]. BsAbs feature two binding sites that simultaneously recognize two distinct antigens or two different epitopes on the same antigen. The concept is that BsAbs would exhibit stronger antitumor activity. Cadonilimab, a tetravalent anti-PD-1/anti-CTLA4 BsAb, is being investigated in GAC both in localized and advanced settings [19,20,21]. Cadonilimab has PD-1 and CTLA-4 binding arms along with Fc silencing [19]. The result is simultaneous cis binding when PD-1 and CTLA-4 co-occur on the same T cell or across the T cell–antigen-presenting cell synapse, creating receptor clustering and stronger immune activation, while the Fc silencing can reduce some of the immune-related adverse effects. Although not dedicated to MSI-H/dMMR GAC/GEJAC, these patients can enroll in cadonilimab trials [20,21]. Pooling the outcomes in MSI-H/dMMR GAC will be useful as this area matures. Cadonilimab is also being evaluated for neoadjuvant therapy and in the refractory anti-PD-1 therapy setting in dedicated MSI-H/dMMR colorectal cancer (CRC) trials [22,23]. ADCs are increasingly being directed at any surface protein or peptide that is highly prevalent in cancer cells compared to normal cells. Therefore, ADCs could also play an increasing role in relapsing MSIH tumors. Most advancements in MSI-H/dMMR are often determined first in CRC as this malignancy is seen more commonly and has a higher percentage of MSI-H/dMMR patients. We look forward to seeing advancements in these areas, along with watch-and-wait strategies using immune checkpoint therapies [24,25], so that these can be employed in the GAC setting. Additionally, other BsAbs are in development that target PD-1 and LAG3 [19]. This could be another area for exploration in MSI-H/dMMR patients.

### 2.2. HER2+ GAC: Current Treatments and Future Outlooks

#### 2.2.1. First-Line Therapy

HER2 has been an oncology molecular target for decades [26,27]. It is expressed in many malignancies, including GAC. However, HER2 overexpression and how patients respond to HER2-directed therapy have been a work in progress with promising results. For GAC, trastuzumab, a humanized monoclonal antibody (mAb) that binds to the extracellular domain IV, has been utilized in upfront advanced HER2+ GAC since the results of the 2010 ToGA trial [28]. ToGA, a phase 3 trial, compared standard of care (SOC) chemotherapy with fluoropyrimidine plus cisplatin to SOC chemotherapy with trastuzumab. Outcomes were improved: median overall survival (OS) of 13.8 months compared to 11.1 months, *p* = 0.0046; median progression-free survival (PFS) of 6.7 months compared to 5.5 months, *p* = 0.0002. The combination of trastuzumab with upfront fluoropyrimidine plus platinum therapy remains SOC to this day [1]. This remained SOC as other front-line trials, such as the two phase 3 trials JACOB and LOGiC, failed to advance HER2-directed therapy [29,30]. JACOB looked at adding pertuzumab, an anti-HER2 mAb that binds to extracellular domain II, to trastuzumab plus front-line chemotherapy compared to trastuzumab plus chemotherapy alone [29]. The primary endpoint was not met (median OS 17.5 months vs. 14.2 months, *p* = 0.057), and other endpoints were not significantly improved (median PFS 8.5 months vs. 7 months). LOGiC evaluated lapatinib, a HER2 and epidermal growth factor receptor (EGFR) tyrosine kinase inhibitor [30]. Lapatinib was combined with front-line chemotherapy compared to chemotherapy alone. The primary endpoint was not met (median OS 12.2 months vs. 10.5 months, *p* = 0.3492). Additionally, the median PFS was not statistically improved (median PFS 6 months vs. 5.4 months, *p* = 0.381). The failures seen with HER2-therapy-directed trials in advanced GAC are likely multifactorial. Intratumor and interpatient HER2 overexpression heterogeneity along with loss of HER2 expression are likely reasons for trial setbacks [31].

Until 2025 and the results of KEYNOTE-811, no additional treatment advances were seen in the front-line advanced HER2+ GAC space [32,33]. KEYNOTE-811, a phase 3 trial, evaluated the role of anti-PD-1 therapy with pembrolizumab added to front-line trastuzumab with chemotherapy (fluoropyrimidine plus platinum). In patients with a PD-L1 CPS ≥ 1, median PFS and median OS were improved (median PFS 10.9 months vs. 7.3 months, HR 0.72; 95% CI 0.60–0.87; median OS 20.1 months vs. 15.7 months, HR 0.79; 95% CI: 0.66–0.95). These improvements are marginal. A limited number of patients had PD-L1 CPS < 1 and fared poorly compared to controls (pembrolizumab is not recommended for these patients). Therefore, the current SOC for those with PD-L1 CPS ≥ 1 is to add pembrolizumab to trastuzumab plus fluoropyrimidine and platinum therapy in the front-line HER2+ advanced GAC setting [1].

Changes to front-line therapy are likely to be on the horizon with the positive results of exploring zanidatamab (ZW25) [34]. Zanidatamab received FDA approval for HER2+ advanced cholangiocarcinoma in 2024 [35]. Zanidatamab is a biparatopic HER2 BsAb that binds to two extracellular domains (IV and II) [36]. Zanidatamab binds to sites targeted by trastuzumab and pertuzumab mAbs, respectively, but zanidatamab binds these HER2 receptors in trans, creating HER2 receptor clustering. This clustering leads to HER2 internalization, downregulation, and potent complement-dependent cytotoxicity (CDC). The CDC impact differs from trastuzumab and pertuzumab. Additionally, it inhibits cell signaling and tumor growth via antibody-dependent cellular cytotoxicity (ADCC) and phagocytosis (ADCP). It has shown superiority in vivo antitumor activity compared to trastuzumab and pertuzumab.

Zanidatamab activity in advanced HER2+ GAC has been exciting. NCT03929666, a phase 2 multicenter global trial (USA, Canada, Korea, and South America), involved treating patients with advanced gastroesophageal adenocarcinoma (GEAC) with HER2+ (IHC 3+ or 2+ with or without gene amplification) [37]. Zanidatamab was given front-line with fluoropyrimidine plus platinum (capecitabine + oxaliplatin, fluorouracil + oxaliplatin, or fluorouracil + cisplatin). The median follow-up reported was 41.5 months. The overall response rate (ORR) was 84%, with a median duration of response of 18.7 months and median PFS of 15.2 months. The estimated 24-month OS rate was 65%. Antidiarrheal prophylaxis was added after 25 patients were treated. HERIZON-GEA-01 was the phase 3 global (although not in the USA) randomized trial of zanidatamab with chemotherapy (capecitabine + oxaliplatin or fluorouracil + cisplatin) +/− tislelizumab, and anti-PD-1 agent, or trastuzumab + chemotherapy [34]. At the start of this trial, trastuzumab plus chemotherapy was still the SOC in this setting. HER2+ (majority IHC 3+) advanced GEAC patients were randomized 1:1:1: trastuzumab + chemotherapy (*n* = 308), zanidatamab + chemotherapy (*n* = 304), and zanidatamab + tislelizumab + chemotherapy (*n* = 302). The treatment cycles were every 3 weeks. Zanidatamab was dosed at a 1800 mg flat dose for those <70 kg or 2400 mg for those ≥70 kg IV every 3 weeks. The median follow-up was 26 months. Compared with the trastuzumab plus chemotherapy arm, both zanidatamab groups did significantly better. The median PFS was 8.1 months in the trastuzumab plus chemotherapy group, while it was 12.4 months in the zanidatamab arms (*p* < 0.0001). The median OS was 19.2 months in the trastuzumab plus chemotherapy arm compared to 24.4 months in the zanidatamab plus chemotherapy arm and 26.4 months in the group receiving the addition of tislelizumab. The final publication has not been released to date, but, with these preliminary report findings, zanidatamab is likely to change practice with these results. Issues that will need to be worked out clinically are the management of diarrhea along with the toxicity of the chemotherapy regimens utilized in this study as capecitabine can often be difficult for GEAC patients to handle due to dysphagia and 14-day-on/7-day-off dosing toxicity. Additionally, cisplatin as the platinum utilized with 5-fluorouracil is a somewhat dated practice given the toxicity seen with cisplatin-containing regimens over oxaliplatin-based regimens in GEAC.

The superior drug design of zanidatamab along with the improvements seen translationally and clinically will replace the traditional approach of trastuzumab with chemotherapy in the front-line setting for advanced HER2-positive GEAC. The addition of tislelizumab, regardless of PD-L1 status, will also allow all HER2+ GEAC patients without uncontrolled autoimmune contraindications to receive immune checkpoint therapy. Diarrhea, being a significant adverse effect that is notably higher, would not hinder a practice change from HER2 mAb inhibition to HER2 BsAb inhibition. Diarrhea can be managed and appears to become tolerable with time.

#### 2.2.2. Other HER2-Directed Therapy

BsAbs are also being converted to ADCs, and this could bring another therapeutic dimension for solid tumors. Trastuzumab deruxtecan, an antibody–drug conjugate (ADC), consists of trastuzumab with a topoisomerase inhibitor payload [38]. ADCs are designed to deliver cytotoxic payloads to tumor cells with the idea of achieving higher tumor specificity while minimizing off-target toxicity. The composition involves an mAb that binds selectively to tumor-associated antigens or overexpressed proteins. The mAb is covalently linked to the cytotoxic payload. The success in ADC design likely depends on the consistency of the target, as discussed by Matsuda et al. in the differences in success between trastuzumab deruxtecan (HER2 targeting in HER2+ breast cancer and GAC/GEAC) and patritumab deruxtecan (HER3 targeting in EGFR-mutated non-small-cell lung cancer).

Trastuzumab deruxtecan was the first FDA-approved approach for second-line therapy in advanced HER2+ GAC in 2021 [39]. FDA approval was a result of the DESTINY-Gastric 01 trial, a phase 2 Asian trial [40]. Trastuzumab deruxtecan (*n* = 125) was compared to physician choice chemotherapy with paclitaxel or irinotecan (*n* = 62). OS was improved with trastuzumab deruxtecan, showing a median OS of 12.5 months vs. 8.4 months, *p* = 0.01). The median PFS was also improved (5.6 months vs. 3.5 months). DESTINY-Gastric-02, a phase 2 single-arm trial in the USA and Europe, examined trastuzumab deruxtecan in Western populations [41]. Similar outcomes were seen, with a median OS of 12.1 months and a median PFS of 5.6 months. DESTINY-Gastric-04 was a phase 3 international trial comparing trastuzumab deruxtecan (*n* = 246) and current SOC ramucirumab plus paclitaxel (*n* = 248) in those HER2+ GAC patients who progressed on front-line trastuzumab-based therapy [42]. Trastuzumab deruxtecan proved to be superior in OS (median OS 14.7 months vs. 11.4 months, *p* = 0.004), with minor changes in PFS (median PFS 6.7 months vs. 5.6 months, *p* = 0.007). Given these results, trastuzumab deruxtecan remains the second-line therapy after trastuzumab front-line HER2 GAC progression [1]. Tolerability concerns with this agent do exist and are seen in the clinic with emesis, myelosuppression, diarrhea, and interstitial pneumonitis. Therefore, candidates for trastuzumab deruxtecan must have good performance status prior to starting. Front-line phase 3 trials with trastuzumab deruxtecan in combination therapy are enrolling (DESTINY-Gastric-05 and Artemide Gastric01) [43,44]. Study completions are anticipated in 2030. These phase 3 front-line trials were designed prior/simultaneously to zanidatamab front-line exploration, so the results would not factor in or directly compare the potential change in front-line therapy with zanidatamab. Efficacy results would have to balance the significantly higher toxicity of trastuzumab deruxtecan for it to move placement to front-line.

Of notable interest, and where we would like to see expanded data, is the use of HER2 ADCs like trastuzumab deruxtecan in those with HER2-low expression (IHC 2+/FISH negative; IHC 1+). Efficacy promise has been reported in exploratory cohorts, like from the refractory population in DESTINY-Gastric-01 (ORR 9.5% and median PFS 2.8 months for IHC 1+; ORR 26.3% and median PFS 4.4 months for IHC 2+/FISH negative) [40]. HER2 mAbs have shown to be consistently less impactful in HER2-low disease compared to HER2 IHC 2+/FISH-positive and IHC 3+ GAC/GEJAC patients. Additionally, zanidatamab appears to be the most impactful for IHC3+ GAC/GEJAC patients. We need therapies that could be efficacious in those with lower HER2 positivity as these patients may not have any targeted options and particularly none in the refractory setting. ADCs have that potential advantage and could provide an option for those currently without HER2-targeted treatment options.

Another HER2 ADC showing promise in advanced HER2 GAC is disitamab vedotin (RC48). Disitamab vedotin is an anti-HER2 mAb. Disitamab is thought to have higher binding affinity than trastuzumab, with monomethyl auristatin E (MMAE) as the payload. It is showing promise in the HER2+ low setting (mostly IHC1+), which makes this an interesting molecule to monitor in upcoming years as more data emerges [45]. Shen et al. reported an ORR range of 48–77% with median PFS 5.6–9.7 months, dependent on the cohort, with front-line disitamab vedotin combined with anti-PD-1 therapy and chemotherapy. Phase 3 trials are underway [46,47]. The MMAE payload, however, does cause concern for neuropathy, which is commonly seen with front-line platinum chemotherapy and second-line options in advanced GAC/GEJAC. HER2-low is an unmet need for both front-line and refractory GAC/GEJAC. We hope to see expanded research regarding HER2 ADCs for this subgroup.

HER2 targeting has been an important marker in GAC/GEJAC and one with staying power. Advancements and investigation will remain central regarding this malignancy. Understanding HER2 loss and heterogeneity will lead to further advancements. Hopes of circulating tumor DNA advancements might aid in patient selection and clinically understanding the changes in HER2 as a patient’s disease progresses. Relying on tissue-based testing continues to hinder progress as repeat biopsies are not often performed clinically.

### 2.3. CLDN18.2: Current Treatments and Future Outlooks

#### 2.3.1. First-Line Therapy

Claudins (CLDNs) encompass a family of 27 transmembrane proteins that are essential components of tight junctions in epithelial and endothelial cells [48,49,50,51,52,53]. CLDNs help to maintain paracellular permeability, cell polarity, and cell-to-cell adhesions. Unique to this new target is the identification that each CLDN has a tissue- and organ-specific pattern along with differences in expression between normal cells and malignant cells. CLDN18 has two variants, CLDN18.1 and CLDN18.2. CLDN18.2 is predominately seen in gastric epithelial cells. Malignant transformation allows exposure to CLDN18.2 on the cell surface, allowing for a drug binding target, leading the way for zolbetuximab, a chimeric mAb, that binds to CLDN18.2. Approved by the FDA in 2024, zolbetuximab is the first in this class of agents and represents a new option for advanced GAC/GEJAC CLDN18.2-positive patients [10]. Early trial development clarified the CLDN18.2 expression level required for zolbetuximab’s use (CLDN18.2 ≥ 75% in tumor cells with 2+ or 3+ expression intensity). Through two phase 3 trials, SPOTLIGHT and GLOW, zolbetuximab combined with front-line chemotherapy (fluoropyrimidine plus platinum) showed improvement compared to SOC chemotherapy (SPOTLIGHT: median OS 18.2 months vs. 15.5 months, *p* = 0.0053 and median PFS 10.6 months vs. 8.7 months; GLOW: median OS 14.3 months vs. 12.1 months, *p* = 0.0118 and median PFS 8.2 months vs. 6.8 months, *p* = 0.0007) [11,12]. ORR was not impacted by zolbetuximab’s addition. Zolbetuximab with SOC chemotherapy is a current front-line option for advanced GAC/GEJAC patients with CLDN18.2 expression [1]. However, toxicity concerns, particularly emesis, are present with this agent, and logistically long infusion times are seen in the clinic. Management of these issues is still being worked out to improve patient quality of life while on this agent.

#### 2.3.2. Future Directions

The identification of a new target is exciting in advanced GAC/GEJAC, which, as mentioned previously, has not had as many robust molecular targets as other solid-tumor malignancies. Given this identification, the CLDN18.2 market is heavily saturated with compounds such as other mAbs, BsAbs, ADCs, and chimeric antigen receptor T cell therapy. BsAb examples include givastomig, IBI389, AZD5863, ASP2138, QLS31905, and PT886, to name a few [54,55,56,57,58,59]. Currently, these are all in early clinical development, but we anticipate knowledge gain in this area in the near future. There are also a plethora of ADCs in development, with some in phase 3 trials, including LM-302, IBI-343, and AZD0901 [60,61,62]. LM-302, an anti-CLDN18.2 mAb with an MMAE payload, was recently investigated for the refractory setting of advanced GAC/GEJAC compared to physician choice of apatinib or irinotecan [60]. Study completion is anticipated in 2026. IBI-343, an anti-CLDN18.2 ADC utilizing a topoisomerase inhibitor as the payload, is currently in a phase 3 trial evaluating its role in previously treated CLDN18.2-positive advanced GAC/GEJAC patients [61]. Study completion is expected in 2027. AZD0901 utilizes an MMAE payload and is also being evaluated in the phase 3 refractory setting for advanced CLDN18.2 GEAC [62]. Study completion is estimated for 2026.

The market is robust for CLDN18.2 in the GAC/GEJAC setting [63]. We anticipate many changes in this area, including targeting those with lower CLDN18.2 expression with more development, particularly for BsAbs and ADCs in this area. Understanding the pathophysiology and mechanism behind the emesis induced by targeting CLDN18.2 will need to be better understood as progress continues in this area. The cumbersome logistics, high toxicity, and only marginal improvement seen with zolbetuximab create the need for improved targeting of CLDN18.2. We look forward to better drug design in this area.

### 2.4. Trophoblast Cell-Surface Antigen 2

#### Potential Future

Trophoblast cell-surface antigen 2 (TROP-2), a cell-surface glycoprotein (a Do Not Eat Me signal) found on epithelial cells, has, in recent years, become a target for solid tumors [64]. Two ADCs are currently FDA approved—sacituzumab govitecan and datopotamab deruxtecan—for other solid tumors [65,66]. Both are anti-TROP2 mAbs with topoisomerase inhibitor payloads. Additionally, there are several other anti-TROP2 ADCs in development, including sacituzumab tirumotecan, SHR-A1921, DB1305, and OBI-902, to name a few [67,68,69,70]. BsAbs are also in development with F7Ak3, AVZO-103, and TROP2/CD3 engagers [71,72,73]. However, these are mostly translational pre-clinical, so it is too early to extrapolate their use in GAC/GEJAC. AVZO-103 is the furthest along, with phase 1/2 evaluation in other solid tumors. With multiple agents in the TROP2 pipeline, cross-resistance will need to be studied further.

Overexpression in GAC/GEJAC has been noted (>50%) [64], and, with drug development currently existing in this area, how these agents will fare in GAC/GEJAC needs to be clarified. Another biomarker-driven therapy would be beneficial in mGAC. TROP2 overexpression is much higher than current biomarker-driven therapies, like MSI-H/dMMR, HER2, PD-L1 CPS, and CLDN18.2. Having agents targeting many GAC/GEJAC cases would of course be beneficial.

Currently, data in this area is still in early development, but we look forward to continued data reporting with trials like TroFuse-015, a phase 3 trial evaluating sacituzumab tirumotecan in the refractory advanced (≥3 lines of therapy) GAC/GEJAC setting [67]. TroFuse-015 outcomes will be of considerable interest given the lack of impactful non-biomarker-driven options in this high-refractory setting, like trifluridine–tipiracil. The estimated completion is in 2027. Early phase 1/2 data in the GAC/GEJAC cohort showed an ORR of 22%, a DCR of 80%, and a median PFS of 3.7 months [74]. The SAGA trial is exploring sacituzumab govitecan in GEAC after at least one line of therapy and should be completed this year [75]. Datopotamab deruxtecan in combination with fluoropyrimidine therapy is currently enrolling in TROPION-PanTumor03, a phase 2 solid-tumor trial [76]. Looking at the trifluridine–tipiracil outcomes (the ORR was 4%, with a median PFS of 2 months, which is only a 0.2-month improvement compared with a placebo) [77], TROP2 targeting looks promising as a refractory therapy option and should continue to be an investigative priority.

## 3. Conclusions

Biomarker-driven therapy has come a long way in GAC/GEJAC treatment. The treatment of MSI-H/dMMR disease has been revolutionized by immune checkpoint therapy. Unfortunately, there are still subsets of patients who do not respond. Therefore, progress in drug development with BsAbs like cadonilimab could potentially help to decrease the percentage of non-responders. A change in front-line HER2 therapy is anticipated this year given the results seen with zanidatamab. Most patients in HERIZON-GEA-01 had HER2 IHC 3+; therefore, understanding how zanidatamab with chemotherapy will fare efficacy-wise in those who are HER2 IHC 2+/FISH-positive remains an aspect to be elucidated in clinical practice. Additionally, the efficacy of trastuzumab deruxtecan after zanidatamab progression will be key to report on in the coming years. Development for HER2-low patients with ADCs will continue to be monitored, with the hope of potential targeting in this population. More sophisticated drug technology is needed to improve upon the CLDN18.2 targeting seen with zolbetuximab. Trials are underway in all these areas, with potential advancements (Table 1) including additional targets that are not yet directed at GAC, like TROP-2. Many BsAbs and ADCs are currently being investigated in these malignancies, which is likely to facilitate changes in their management in the coming years. Examples of BsAbs and ADCs are listed in Table 2 [78,79,80,81]. We look forward to these advancements.

## Figures and Tables

**Table 1:** Examples of phase 3 trials in advanced gastroesophageal adenocarcinoma [18,31,38,39,41,42,54,56,60].

Trial	Treatment	Population	Primary Endpoint and Estimated Completion Date
**MSI-H/dMMR**			
**NCT06346197**	Botensilimab + balstilimab vs. chemotherapy + nivolumab	Firstline advanced MSI-H/dMMR GEAC	Overall survival 2028
		**HER2+**	
**HERIZON-GEA-01**	Zanidatamab + chemotherapy +/− tislelizumab vs. trastuzumab + chemotherapy	Firstline advanced HER2+ GEAC	Progression-free survival and overall survival completed
**NCT06731478**	Trastuzumab deruxtecan + pembrolizumab + chemotherapy vs. trastuzumab + pembrolizumab + chemotherapy	Firstline advanced HER2+ GEAC	Progression-free survival 2030
**NCT06764875**	Rilvegostomig + trastuzumab deruxtecan + fluoropyrimidine vs. pembrolizumab + trastuzumab + chemotherapy vs. rilvegostomig + trastuzumab + chemotherapy	Firstline advanced HER2+ GEAC	Progression-free survival and overall survival 2030
**NCT06944496**	Disitamab vedotin + tislelizumab + chemotherapy vs. tislelizumab + chemotherapy	Firstline advanced HER2-low GEAC	Progression-free survival 2030
**NCT06221748**	Disitamab vedotin + cadonilimab	Refractory advanced HER2+ GEAC	Objective remission rate, adverse events, progression-free survival and overall survival 2027
**CLDN18.2**			
**NCT06351020**	LM-302 vs. physician choice therapy	Refractory advanced CLDN 18.2+ GEAC	Progression-free survival and overall survival 2026
**NCT06346392**	AZD0901 vs. physician choice therapy	Refractory advanced CLDN18.2+ GEAC	Progression-free survival and overall survival 2026
		**TROP2**	
**TroFuse-015**	Sacituzumab tirumotecan vs. physician choice therapy	Refractory advanced GEAC	Overall survival 2027

MSI-H/dMMR: microsatellite instability-high/deficient mismatch repair; GEAC: gastroesophageal adenocarcinoma; HER2: human epidermal growth factor receptor-2; CLDN18.2: claudin 18.2; TROP2: trophoblast cell-surface antigen 2.

**Table 2:** Examples of bispecific antibodies and antibody–drug conjugates under investigation for HER2 and CLDN18.2 [78,79,80,81].

HER-2 Bispecific Antibodies Zanidatamab Zenocutuzumab
**HER2 Antibody–Drug Conjugates** Trastuzumab Deruxtecan Disitamab Vedotin MRG002 ARX788 ZW49 SBT6050 GQ1001
**CLDN 18.2 Bispecific Antibodies** Givastomig IBI389 AZD5863 ASP2138 QLS31905 PT886 PM1032 SG1906
**CLDN 18.2 Antibody–Drug Conjugates** LM-302 IBI-343 AZD0901 SHR-A1904 SKB315 JS107 TORL-2-307-ADC

HER2: human epidermal growth factor receptor-2; CLDN18.2: claudin 18.2.

## Data Availability

No new data were created or analyzed in this study.
